# Evaluation of the incidence of congenital uterine anomalies in polycystic ovarian syndrome: tertiary center experience

**DOI:** 10.3389/fmed.2025.1582100

**Published:** 2025-05-22

**Authors:** Onur Yavuz, Aslı Akdöner, Kadir Alper Mankan, Kadircan Gündoğan, Recep Emre Okyay, Ömer Erbil Doğan

**Affiliations:** Department of Obstetrics and Gynecology, Dokuz Eylül University School of Medicine, Izmir, Türkiye

**Keywords:** anti-Müllerian hormone, infertility, polycystic ovary syndrome, septate uterus, uterine abnormality

## Abstract

**Objectives:**

The primary aim of the study was to compare the incidence of congenital uterine anomalies in polycystic ovarian syndrome (PCOS) patients with the control group.

**Methods:**

This was a retrospective cohort study conducted at a tertiary center between January 2018 and January 2024. The study cohort included 297 patients, comprising 99 women with PCOS (PCOS group, 33.3%) and 198 healthy women whose partners had male factor infertility (control group, 66.7%). The uterine cavity was evaluated using hysterosalpingography (HSG) images according to the European Society of Human Reproduction and Embryology (ESHRE) and the European Society for Gynaecological Endoscopy (ESGE) consensus on the classification of female genital tract congenital anomalies and the American Society for Reproductive Medicine (ASRM) Müllerian anomalies classification guidelines. Demographic characteristics, physical examination findings, laboratory results, and HSG findings of the groups were compared. Analyses were performed with SPSS version 26.0. Variables that did not show a normal distribution were analyzed using the Mann–Whitney U-test. The chi-squared test and Fisher exact test were used to analyze categorical data. An inter-rater reliability analysis (Cohen’s kappa) was performed for HSG findings. The results were reported with a 95% confidence interval (CI). The *p*-value of <0.05 was considered statistically significant.

**Results:**

Of the whole study cohort, 7.7% had congenital uterine anomalies (CUAs) according to the ASRM criteria and 4.7% had CUAs according to the ESHRE/ESGE classification. CUAs were 5.7 times higher in the PCOS group than in the control group according to the ASRM criteria and 5.5 times higher in the PCOS group than the control group according to the ESHRE/ESGE classification system (17.2% vs. 3%, *p* < 0.0001; 10.1% vs. 2%, *p* = 0.003, respectively). Partial septate uterus (ASRM and ESHRE/ESGE classifications) was the most frequently detected CUA in the PCOS group (9.1% vs. 1.5%, *p* = 0.003). According to the ASRM classification, the partial septate uterus was followed by the arcuate uterus. It was 4.7 times more common in the PCOS group (7.1% vs. 1.5%, *p* = 0.01).

**Conclusion:**

We found that the frequency of CUA was higher among PCOS patients. Prospective studies are needed to examine anti-Müllerian hormone (AMH), serum sex steroids, and pregnancy complications in more detail to clarify pathophysiology and clinical implications.

## Introduction

Polycystic ovarian syndrome (PCOS) is the most common and well-studied endocrine disorder among women of reproductive age, affecting approximately 5–10% of the population. It is characterized by a range of clinical phenotypic presentations (1). Hyperandrogenism, ovulatory dysfunction, and polycystic ovary morphology are among the main features of this syndrome (2, 3). It has been reported that the incidence of pregnancy complications, particularly preterm birth, is higher in PCOS patients compared to normo-ovulatory women.

Congenital uterine anomalies (CUAs) result from defects in the embryological development of the Müllerian ducts. These CUAs lead to decreased pregnancy rates and increased risks of miscarriage and preterm birth (4–6). Genetic, sporadic, or multifactorial factors are believed to contribute to the formation of these Müllerian duct (MD) anomalies.

Studies in the literature have highlighted a strong correlation between the formation and differentiation of the MD and the *Hox* and *Wnt* genes (7–16). Fusion defects of MD, such as uterine septum and didelphys uterus, have been detected in women with HOXA10 mutations (17, 18). Additionally, evidence suggests that these genes are regulated by intrauterine androgens and anti-Müllerian hormone (AMH) (17–19). Moreover, high intrauterine concentrations of AMH reduce the effect of the placental aromatase enzyme, exposing the fetus to higher androgen levels (20). These findings suggest a potential association between PCOS, defects in uterine resorption, and pregnancy complications (17–19).

There are relatively few articles addressing this important topic in the existing literature (21–24). Our study was initiated based on the observation of variations in uterine cavity structure identified through hysterosalpingography (HSG) imaging during routine infertility assessments of patients diagnosed with PCOS. We hypothesized that the incidence of abnormal uterine cavities is greater among patients with PCOS than those without the condition. The primary aim of the study was to compare the incidence of CUA among PCOS patients and the control group.

## Materials and methods

This was a retrospective cohort study conducted at a tertiary center. Informed consent was obtained from all individual participants included in this study. The study was performed according to the principles of the Declaration of Helsinki. Institutional ethics committee provided approval for this study (Registration number: 2024/19–12; Date:29/05/2024).

**Figure 1:**
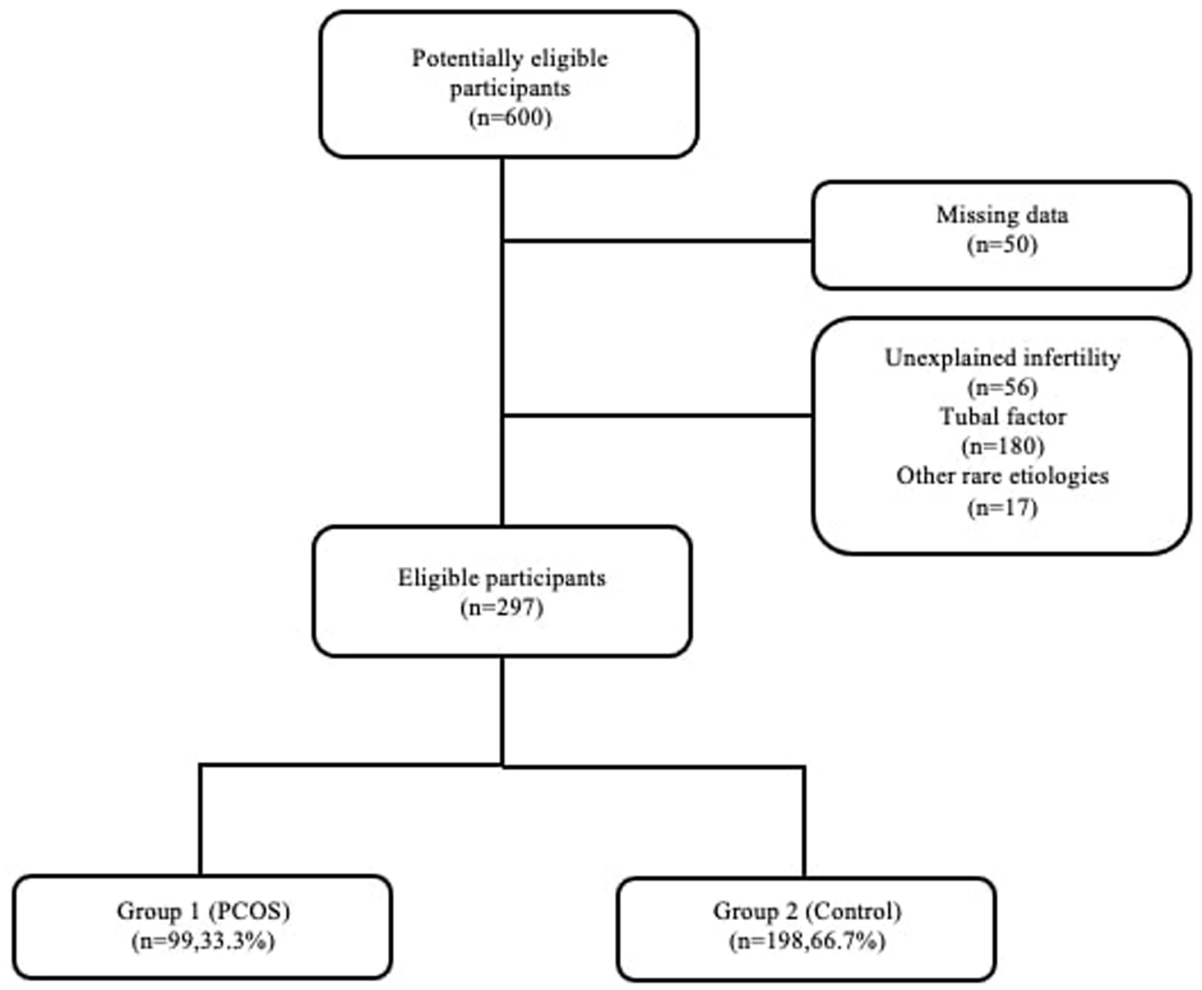
Flow chart of the study.

Between January 2018 and January 2024, demographic parameters and HSG images of 600 patients admitted to our infertility clinic were retrospectively retrieved from the hospital database. Patients with missing demographic or laboratory data and those lacking HSG images were excluded from the study. Moreover, individuals with unexplained infertility without PCOS (*n* = 56, 10.1%), tubal factor infertility (*n* = 180, 32.7%), and other rare etiologies (*n* = 17, 3%) were not included. The final analysis involved 297 patients, comprising 99 classified as PCOS patients, forming the study group (Group1; 33.3%), and 198 healthy women whose partners had male factor infertility, constituting the control group (Group 2; 66.7%) (Figure 1).

PCOS patients were diagnosed with the Rotterdam Criteria, which require the presence of at least two of the following: oligo/anovulation, clinically or laboratory-confirmed hyperandrogenism, and polycystic ovaries on ultrasound, after exclusion of other causes of hirsutism (25). The morphology of the polycystic ovary was defined as an ovary containing 12 or more follicles with a diameter of 2–9 mm and/or an increase in ovarian volume (>10 cm^3^), as detected by ultrasonography. During clinical examination, hirsutism was evaluated according to the Ferriman-Gallwey (FG) scoring system (26). Patients with a score above eight were considered to have hirsutism. The basal serum hormone levels were examined between the second and fifth days of the menstrual cycle. All HSG images were numbered and filed for each patient. The HSG images of patients were numbered and filed by an independent person blinded to the research. The uterine cavity was evaluated according to the European Society of Human Reproduction and Embryology (ESHRE), the European Society for Gynaecological Endoscopy (ESGE) consensus on the classification of female genital tract congenital anomalies, and the American Society for Reproductive Medicine (ASRM) Müllerian anomalies classification guidelines (27, 28). All HSG images were independently and retrospectively evaluated by two reviewers (OY and AA, respectively), each with over 5 years of experience in reproductive endocrinology and surgery. In case of discrepancies, a third reviewer with over 20 years of experience in reproductive endocrinology and surgery ensured consensus (OED). Demographic parameters, laboratory findings, and HSG results were compared between the groups.

Analyses were performed with SPSS version 26.0 (IBM Inc., Chicago, IL, USA). A normality analysis was performed according to the Kolmogorov–Smirnov test. Variables that did not show a normal distribution were analyzed using the Mann–Whitney U-test. These results were expressed as median (minimum-maximum) values for each group. The chi-squared test and the Fisher exact test were used to analyze categorical data. These were presented as counts and percentages (%). An inter-rater reliability analysis was performed for HSG findings. For this purpose, Cohen’s Kappa was calculated and classified as follows: *k* = 0–0.20, slight agreement; *k* = 0.21–0.40, fair agreement; *k* = 0.41–0.60, moderate agreement; *k* = 0.61–0.80, substantial agreement; and *k* = 0.81–1.00, which is a near perfect agreement. The results were reported with a 95% confidence interval (CI). The *p*-value of <0.05 was considered statistically significant.

## Results

Demographic characteristics, physical examination findings, and laboratory results of the groups are listed in Table 1. The ages of the patients included in the study were between 18 and 42 years (median 29 years). There was no difference between the median ages of the groups (29 vs. 30, *p* = 0.2). Although the body mass index (BMI) of the PCOS group was found to be higher, the groups were similar (25.9 vs. 25.7, *p* = 0.9). As expected, the hirsutism rate and FG score were statistically significantly higher in the PCOS group (95.1% vs. 4.9%, *p* < 0.0001; 12 vs. 5, *p* < 0.0001, respectively). Oligomenorrhea/amenorrhea was 5.3 times more common in the PCOS group (53.3% vs. 10.1%, *p* < 0.0001). The ratio of luteinizing hormone (LH) to follicle-stimulating hormone (FSH) (LH/FSH) was statistically higher in the PCOS group (0.9 vs. 0.7, *p* < 0.0001).

**Table 1:** Demographic characteristics, physical examination findings and laboratory results of groups.

Variables	All patients (*n* = 297,100%)	Group 1 (PCOS) (*n* = 99, 33.3%)	Group 2 (Control) (*n* = 198, 66.7%)	*p* value
Age (years)	29 (18–42)	29 (21–42)	30 (18–42)	0.2
BMI (kg/m^2^)	25.7 (16–39.8)	25.9 (16–29.8)	25.7 (16.6–38.9)	0.9
Hirsutism (n,%)	81/297 (27.3%)	77/99 (95.1%)	4/198 (4.9%)	<0.0001
FG score	6 (2–28)	12 (3–28)	5 (2–12)	<0.0001
Primary infertility (n,%)	265/297 (89.2%)	90/99 (90.9%)	175/198 (88.4)	0.5
Oligomenorrhea/amenorrhea (n,%)	73/297 (24.6%)	53/99 (53.5%)	20/198 (10.1%)	<0.0001
Duration of infertility (years)	3 (1–21)	3 (1–12)	3.2 (1–21)	0.9
FSH (IU/L)	6.9 (3.4–9.9)	6.3 (3.4–9.4)	7.3 (3.5–9.9)	<0.0001
LH (IU/L)	5.5 (1.3–22.7)	6 (1.3–22.7)	5.3 (2.1–19)	<0.0001
LH/FSH	0.7 (0.2–3.9)	0.9 (0.2–3.9)	0.7 (0.2–3.1)	<0.0001
E2 (pg/mL)	50 (11.5–185)	43 (17–166)	50 (11.5–173.5)	0.01

PCOS, Polycystic ovarian syndrome; BMI, Body mass index; FG, Ferriman-Gallwey; FSH, Follicle stimulating hormone; LH, Luteinizing hormone; E2, Estradiol.

Interobserver agreement in evaluating the uterine cavity with hysterosalpingography according to the classifications is shown in Table 2. The interobserver agreement was nearly perfect for the normal uterus (ASRM classification), didelphys uterus (ASRM classification), and partial septate uterus (ASRM and ESHRE/ESGE classifications). There was a substantial agreement for arcuate uterus (ASRM classification), normal uterus (ESHRE/ESGE classification), dysmorphic uterus (ESHRE/ESGE classification), and partial bicorporeal uterus (ESHRE/ESGE classification).

**Table 2:** Interobserver agreement in the evaluation of the uterine cavity with hysterosalpingography according to classifications.

Variables	Interobserver agreement rate (%)	Kappa [CI 95%]	*p* value
ASRM classification			
Normal uterus	97.6%	0.83 (0.72–0.95)	<0.0001
Arcuate uterus	98.6%	0.79 (0.59–0.99)	<0.0001
Didelphys uterus	100%	1.00 (1.00–1.00)	<0.0001
Partial septate uterus	99.3%	0.88 (0.72–1.00)	<0.0001
ESHRE/ESGE classification			
Normal uterus	97.9%	0.78 (0.62–0.95)	<0.0001
Partial septate uterus	98.9%	0.86 (0.71–1.00)	<0.0001
Dysmorphic uterus	99.6%	0.66 (0.04–1.00)	<0.0001
Partial bicorporeal uterus	99.6%	0.66 (0.04–1.00)	<0.0001

ASRM, American Society for Reproductive Medicine; ESHRE, European Society of Human Reproduction and Embryology; ESGE, European Society for Gynaecological Endoscopy; CI, Confidence interval.

The comparison of hysterosalpingography results between the groups is given in Table 3. Of the whole study cohort, 92.3% had normal uteri according to ASRM and 95.3% according to the ESHRE/ESGE classification. The PCOS group had a significantly higher rate of CUA (abnormal uteri) according to ASRM and ESHRE/ESGE classifications (17.2% vs. 3%, *p* < 0.0001; 10.1% vs. 2%, *p* = 0.003, respectively). Arcuate uterus (ASRM classification) and partial septate uterus (ASRM classification) were at a statistically significantly higher rate in the PCOS group (7.1% vs. 1.5%, *p* = 0.01; 9.1% vs. 1.5%, *p* = 0.003, respectively). Didelphys uterus (ASRM classification) rate was similar between the groups (1% vs. 0%, *p* = 0.3). The partial septate uterus (ESHRE/ESGE classification) rate was statistically higher in the PCOS group (9.1% vs. 1.5%, *p* = 0.003). The dysmorphic uterus (ESHRE/ESGE classification) and partial bicorporeal uterus (ESHRE/ESGE classification) rates were similar between groups (0% vs. 0.5%, *p* = 0.4; 1% vs. 0%, *p* = 0.3, respectively).

**Table 3:** Comparison of hysterosalpingography results between groups.

Variables	All patients (*n* = 297, 100%)	Group 1 (PCOS) (*n* = 99, 33.3%)	Group 2 (Control) (*n* = 198, 66.7%)	*p* value
ASRM classification				
Normal uterus	274/297 (92.3%)	82/99 (82.8%)	192/198 (97%)	<0.0001
Arcuate uterus	10/297 (3.4%)	7/99 (7.1%)	3/198 (1.5%)	0.01
Didelphys uterus	1/297 (0.3%)	1/99 (1%)	0/198 (0%)	0.3
Partial septate uterus	12/297 (4%)	9/99 (9.1%)	3/198 (1.5%)	0.003
ESHRE/ESGE classification				
Normal uterus	283/297 (95.3%)	89/99 (89.9%)	194/198 (98%)	0.003
Partial septate uterus	12/297(4.1%)	9/99 (9.1%)	3/198 (1.5%)	0.003
Dysmorphic uterus	1/297 (0.3%)	0/99 (0%)	1/198 (0.5%)	0.4
Partial bicorporeal uterus	1/297 (0.3%)	1/99 (1%)	0/198 (0%)	0.3

PCOS, Polycystic ovarian syndrome; ASRM, American Society for Reproductive Medicine; ESHRE, European Society of Human Reproduction and Embryology; ESGE, European Society for Gynaecological Endoscopy.

Table 4 shows the demographic characteristics, physical examination findings, and laboratory results of PCOS patients with either a normal or abnormal uterine cavity according to both classifications. According to the ESHRE/ESGE classification, CUA was detected in 10 of 99 PCOS cases (10.1%). According to the ASRM classification, CUA was detected in 17 of 99 PCOS cases (17.2%). Across both classifications, the characteristics of the PCOS and control groups were similar (*p* > 0.05).

**Table 4:** Demographic characteristics, physical examination findings and laboratory results of PCOS patients with the presence of normal or abnormal uterine cavity in both classifications.

Variables	ESHRE/ESGE classification			ASRM classification		
	Normal (*n* = 89, 89.9%)	Abnormal (*n* = 10, 10.1%)	*p* value	Normal (*n* = 82, 82.8%)	Abnormal (*n* = 17, 17.2%)	*p* value
Age (years)	29 (21–42)	33 (24–39)	0.3	29 (21–42)	29 (24–39)	0.5
BMI (kg/m^2^)	25.8 (16–39.8)	27.8 (17.1–36)	0.5	25.7 (16–39.8)	27.8 (17.1–36)	0.4
Hirsutism (*n*, %)	69/89 (77.5%)	8/10 (80%)	0.8	76/82 (92.7%)	14/17 (82.4%)	0.1
FG score	13 (3–28)	12 (6–25)	0.9	3 (1–11)	4 (1–12)	0.6
Primary infertility (*n*, %)	82/89 (92.1%)	8/10 (80%)	0.2	64/82 (78%)	13/17 (76.5%)	0.5
Oligomenorrhea/amenorrhea (*n*, %)	48/89 (53.9%)	5/10 (50%)	0.8	45/82 (54.8%)	8/17 (47.1%)	0.3
Duration of infertility (years)	3 (1–12)	4 (1–7)	0.5	3 (1–11)	4 (1–12)	0.2
FSH (IU/L)	6.3 (3.4–9.4)	6.2 (3.5–7.5)	0.5	6.3 (3.4–9.4)	6.3 (3.5–9.4)	0.8
LH (IU/L)	5.9 (1.3–22.7)	7.4 (4.8–15.4)	0.1	5.9 (1.3–22.7)	6.8 (1.6–15.4)	0.8
LH/FSH	0.9 (0.2–3.9)	1.3 (0.6–3.2)	0.09	0.9 (0.1–3.9)	1.1 (0.2–0.9)	0.8
E2 (pg/mL)	43 (17–166)	44 (31–77)	0.3	49.9 (17–166)	47 (23.7–83.2)	0.2

PCOS, Polycystic ovarian syndrome; ASRM, American Society for Reproductive Medicine; ESHRE, European Society of Human Reproduction and Embryology; ESGE, European Society for Gynaecological Endoscopy; BMI, Body mass index; FG, Ferriman-Gallwey; FSH, Follicle stimulating hormone; LH, Luteinizing hormone; E2, Estradiol.

## Discussion

In this current study, PCOS patients and control patients whose partners had male factor infertility were compared in terms of CUA. Of the entire study cohort, 7.7% had abnormal uteri according to the ASRM classificationand 4.7% had abnormal uteri according to the ESHRE/ESGE classification. The PCOS group had a significantly higher rate of CUA according to the ASRM and ESHRE/ESGE classifications. Partial septate uterus (classified by both ASRM and ESHRE/ESGE classifications) and arcuate uterus (ASRM classification) were at a statistically significantly higher rate in the PCOS group.

Following the formation of the MD, this structure differentiates into the functional oviduct, uterus, cervix, and upper vagina (17). The complex network of *Hox* and *Wnt* genes ensures the correct formation of the MD (17). Steroid hormones also play a critical role in regulating many of the genes required for proper MD differentiation during organogenesis and adulthood (15). Homeobox genes are required for differentiation and segmental patterning of MD (17). HOXA10 expression is required for the accurate determination of tissue border in the male and female reproductive tracts (17). HOXA10 is present only in the portion of the MD that will differentiate into the uterus (17). Mutations in HOXA10 result in a homeotic transformation of 25% of the proximal uterus into an oviduct (17). Three heterozygous mutations in the HOXA10 gene with a predicted loss of function have been associated with uterine malformations (17, 29).

The Wnt pathway is required for MD patterning and differentiation (30). Wnt7a has an important function in both MD regression in male patients and MD differentiation in female patients (30). Wnt7a is expressed throughout the MD epithelium before birth. After birth, expression is maintained in the oviductal and uterine epithelium but is downregulated in the vaginal epithelium (30). Before the onset of MD regression in male patients, Wnt7a, which signals from the epithelium to the mesenchyme of the MD, activates AMH type II receptor expression in both sexes and ensures proper differentiation of the MD. Because AMH type II receptor expression is lost in the MD mesenchyme in Wnt7a mutant male patients, MD-derived organs are preserved, thus blocking the AMH signaling pathway. Consistent with the differentiation defects observed in the Wnt7a mutant female reproductive system, the ectopic female reproductive system in the mutant male shows no evidence of oviduct coiling. In adult and neonate Wnt7a mutant female patients, the uterus is smaller in length and diameter and the uterine wall is thinner with less smooth muscle (30). The Wnt5a gene is required to develop the caudal region of the MD and glandular genesis (31). Wnt5a null mice have short, coiled uterine horns but lack a cervix and vagina (31).

During male development, mesenchyme-epithelium interactions mediate MD regression to prevent its development into a uterus and the oviduct (31). MD regression requires binding and signal transduction from the transforming growth factor-*β* (TGF-β) family member, AMH, that is secreted from the Sertoli cells of the fetal testis and its type I and II receptors expressed in MD mesenchyme (32–34). The transcription of the AMH gene is directly regulated by multiple factors in the testis-determining pathway (17). In female patients, AMH production by the ovaries begins at birth, although immunohistochemical studies have suggested that this may begin at the end of gestation (35). Assuming that AMH is synthesized by ovarian granulosa cells during the final month of pregnancy, it is not yet secreted into the bloodstream in significant amounts (36). Tata et al. examined AMH levels in a cohort of 63 control pregnant women and 66 subjects with PCOS at gestational weeks between 16 and 19 (37). They revealed significant differences in AMH median values between the two populations, with AMH being higher in the pregnant PCOS group than in the control group (37). To test whether high prenatal AMH exposure could lead to PCOS later in life, the neuroendocrine reproductive characteristics of female offspring were examined (37). AMH has been shown to have a programming effect leading to gestational and perinatal hyperandrogenism and subsequent changes in the hypothalamic–pituitary-gonadal axis and hormone levels of both mothers and offspring (19, 37). In two studies that reached similar conclusions to the findings of Tata et al., it was shown that high AMH levels decreased placental aromatase activity and increased steroidogenic activity in women with PCOS (20, 38).

The expression of the AMH type II receptor has already been identified outside of the gonads, including in the endometrium, breast, prostate, and cervix in humans and in the developing brain and fetal lungs in mice (39). According to the study by Cimino et al., their neuroanatomical data expand these findings by showing that the AMH type II receptor is expressed in several adult brain regions, including the hippocampus, cortex, and hypothalamus (39). Experimental data reveal that AMH directly activates 50–64% of gonadotropin-releasing hormone (GnRH) neurons in a dose-dependent manner. Interestingly, only 50–70% of all GnRH neurons are believed to be involved in the control of pituitary gonadotropin secretion (40, 41). This finding suggests that AMH may indeed be involved in regulating GnRH/LH secretion. It is suggested that the high AMH plasma levels that characterize many PCOS patients may contribute to the hormonal changes observed in PCOS, such as a significant increase in LH secretion (40). High LH pulsatility has been known to be responsible for increased ovarian androgen production by theca cells (39), which may explain two key diagnostic features of PCOS, namely hyperandrogenemia and hirsutism (39). In addition, in PCOS, ovarian hyperandrogenism, hyperinsulinemia due to insulin resistance, and alterations in intraovarian AMH signaling severely affect dominant follicle selection and follicular growth (39). In conclusion, high prenatal AMH levels may trigger the neuroendocrine disorders of PCOS in offspring through GnRH neuron activation (39).

According to the systematic review by Chan et al., the prevalence of uterine anomalies diagnosed by optimal tests was 5.5% in the unselected population, 8.0% in infertile women, 13.3% in those with a history of miscarriage, and 24.5% in those with both miscarriage and infertility (42). Arcuate uterus (3.9%) was most frequently detected anamoly in the unselected population (2.3%), and its prevalence did not increase in high-risk groups. On the other hand, the septate uterus was the most common anomaly in high-risk populations. It is significantly more prevalent (15.4%) in the high-risk population than in the unselected population (2.3%) (42). Ludwin et al. investigated uterine anomalies according to ESHRE/ESGE-2016 (43), ASRM-2016 (44), and Congenital Uterine Malformation by Experts (CUME)-2018 (45) definitions using three-dimensional (3D) ultrasound in high-risk patients with a history of infertility and/or miscarriage (46). According to the above classifications, the incidence of uterine anomalies among the 261 patients comprising the study cohort is 10, 35, and 16%, respectively. The incidence of septate uterus is 5, 31, and 12%, respectively.

Saleh et al. evaluated the infertile population including 3,900 patients in terms of CUA with 3D or 4D ultrasonography according to the American Fertility Society (AFS) classification (23, 47). There were 409 PCOS patients (10.4%), and CUA was detected in 5.2% of all patients and 36.4% of PCOS patients. Among the PCOS group, 66.4% were arcuate uterus, 12.7% were subseptate uterus, and 10% were septate uterus. In another study with a similar methodology, 3,033 infertile patients, 23.4% of whom were PCOS patients, were examined for CUA (24). However, the methods used to diagnose CUA in that study showed heterogeneity. CUA was statistically significantly higher in the PCOS group (8% vs. 3%). In the PCOS group, the arcuate uterus was twice as common (4% vs. 2%) and the septate uterus was six times (3% vs. 0.5%) more common, and this difference was statistically significant. In the congenital uterine abnormalities (CONUTA) study, HSG imaging findings of the uterine cavity of 51 PCOS patients and 52 male factor infertility patients were compared in terms of CUA using the ASRM and ESHRE/ESGE classifications (22). There was a statistically significant higher rate of abnormal uterus in the PCOS group (ASRM classification: 49% vs. 23%; ESHRE/ESGE classification: 33.3% vs. 5.8%). Subseptum, septum, and dysmorphic uterus were statistically higher in the PCOS group according to both CUA classifications. The arcuate uterus was at a similar rate between groups. In our study, 297 patients had abnormal uteruses, 7.7% according to the ASRM classification and 4.7% according to the ESHRE/ESGE classification. Compared to the CONUTA study, the overall rate of CUA in the PCOS group was lower in our study. However, the rate of CUA was higher in the PCOS group compared to the control group in our study. CUA in the PCOS group is 5.7 times higher than the control group according to the ASRM classification and 5.5 times higher than the control group according to ESHRE/ESGE classification. Partial septate uterus (both ASRM and ESHRE/ESGE classifications) was the most frequently detected CUA in the PCOS group. In the ASRM classification, the partial septate uterus was followed by the arcuate uterus. The prevelance of arcuate uterus was 4.7 times higher in the PCOS group compared to the control group.

In our study, we based the diagnosis of PCOS on the criterion of clinically confirmed hyperandrogenism. Hirsutism rate and FG score were statistically higher in the PCOS group. However, when comparing PCOS patients with normal and abnormal uterine cavities, the hirsutism rate and FG score were similar. In a recent study, Tokhunts et al. reported a significant correlation between hyperandrogenism and I-shaped uterus (48). Among the group of women with adrenal and ovarian hyperandrogenism presenting with infertility and recurrent miscarriages, the rate of T-shaped uterus did not differ significantly from that observed in women with other causes of infertility. In the hyperandrogenism group, an I-shaped uterine cavity anomaly was present in 24.3–39.5% of women. Spontaneous miscarriages were detected in 33.3% of PCOS patients with an I-shaped uterus and in 47.1% of congenital adrenal hyperplasia patients with an I-shaped uterus. A positive correlation was found between free testosterone levels and the presence of uterine abnormalities classified according to the ESHRE-ESGE classification in PCOS patients as a subgroup, with a threshold value of 2.025 ng, a sensitivity of 87.5%, and a specificity of 59% in the CONUTA study (22). Researchers explained this positive correlation by suggesting that the resorption process may not have been completed during the intrauterine period and may continue into adulthood (22).

The limitations of our study include its retrospective design and the relatively limited ethnic diversity of the study cohort, which may affect the generalizability of the findings. Laboratory evaluation for hyperandrogenemia was limited, and comprehensive hormonal profiling, including free testosterone, AMH, and DHEA-S levels, was not systematically performed. Outcomes related to assisted reproductive technology were not reported, and a systematic collection of family history regarding PCOS or infertility was lacking, which could have provided further insights into genetic predisposition. Additionally, the exclusive use of static x-ray hysterosalpingography (HSG), although practical and widely available, may lack the diagnostic precision of three-dimensional (3D) ultrasound or MRI in detecting subtle or complex uterine anomalies, representing an important methodological limitation. Three-dimensional ultrasound or MRI, recommended as a first-line diagnostic tool for congenital uterine anomalies by ESHRE/ASRM/ESGE guidelines, was not systematically utilized. Furthermore, the regularity of menstrual cycles, although noted, was not systematically analyzed as a marker of ovulatory function. Additionally, the multivariate analysis, adjusting for potential confounders such as BMI, age, and hormonal profiles, could not be performed due to incomplete retrospective data, which may limit the strength of the observed associations. Future studies with larger, ethnically diverse populations and standardized, comprehensive hormonal and imaging assessments, including multivariate statistical approaches, are warranted to validate and expand upon these findings.

The strengths of our study include its execution in a tertiary center specialized in CUA, the relatively large sample size compared to similar studies in the field, the sufficient number of patients to allow for subgroup analyses, and the use of a standardized imaging protocol aligned with current international classifications.

In conclusion, there are very few studies in the literature that evaluate the complex relationship between PCOS and CUA. We found that the frequency of CUA was higher among PCOS patients. Prospective studies are needed to examine AMH levels, serum sex steroids, and pregnancy complications in more detail to clarify the pathophysiology and clinical implications.

## Data Availability

The raw data supporting the conclusions of this article will be made available by the authors, without undue reservation.
